# Evaluation of the psychometric properties of the London Measure of Unplanned Pregnancy in Brazilian Portuguese

**DOI:** 10.1186/s12884-016-1037-2

**Published:** 2016-08-24

**Authors:** Ana Luiza Vilela Borges, Geraldine Barrett, Osmara Alves dos Santos, Natalia de Castro Nascimento, Fernanda Bigio Cavalhieri, Elizabeth Fujimori

**Affiliations:** 1 Department of Public Health Nursing, University of Sao Paulo School of Nursing, Sao Paulo, Brazil; 2 Department of Clinical Sciences, Brunel University London, Uxbridge, UB8 3PH UK

**Keywords:** Pregnancy, Intention, Unplanned, Psychometric, Measure, Scale

## Abstract

**Background:**

Estimates of unplanned pregnancy worldwide are of concern, especially in low and middle-income countries, including Brazil. Although the contraceptive prevalence rate is high in Brazil, almost half of all pregnancies are reported as unintended. The only source of nationally representative data about pregnancy intention is the Demographic and Health Survey, as with many other countries. In more recent years, however, it has been realized that concept of unintended pregnancy is potentially more complex and requires more sophisticated measurement strategies, such as the London Measure of Unplanned Pregnancy (LMUP). The LMUP has been translated and validated in other languages, but not Portuguese yet. In this study, we evaluate the psychometric properties of the LMUP in the Portuguese language, Brazilian version.

**Methods:**

A Brazilian Portuguese version of the LMUP was produced via translation and back-translation. After piloting, the mode of administration was changed from self-completion to interviewer-administration. The measure was field tested with pregnant, postpartum, and postabortion women recruited at maternity and primary health care services in Sao Paulo city. Reliability (internal consistency) was assessed using Cronbach’s alpha and item-total correlations. Construct validity was assessed using principal components analysis and hypothesis testing. Scaling was assessed with Mokken analysis.

**Results:**

759 women aged 15–44 completed the Brazilian Portuguese LMUP. There were no missing data. The measure was acceptable and well targeted. Reliability testing demonstrated good internal consistency (alpha = 0.81, all item-rest correlations >0.2). Validity testing confirmed that the measure was unidimensional and that all hypotheses were met: there were lower LMUP median scores among women in the extreme age groups (*p* < 0.001), among non-married women (*p* < 0.001) and those with lower educational attainment (*p* < 0.001). The Loevinger H coefficient was 0.60, indicating a strong scale.

**Conclusion:**

The Brazilian Portuguese LMUP is a valid and reliable measure of pregnancy planning/intention that is now available for use in Brazil. It represents a useful addition to the public health research and surveillance toolkit in Brazil.

## Background

Two decades after sexual and reproductive rights definition and the implementation of the Program of Action of the International Conference on Population and Development, there are still many challenges for the full achievement of its goals and objectives. Unplanned pregnancies are one of those challenges. Even considering the differences in measurement scales and populations, estimates of unplanned pregnancy worldwide are of concern [1–3]. Brazil is no different.

In 2006 the Demographic and Health Survey (DHS) in Brazil showed a high contraceptive prevalence rate (80.6 % among married women) coexisting with almost half of all pregnancies being reported as unwanted or mistimed (47.5 %) [4]. The measurement tool of the DHS has been in place since the 1980s and uses the same basic questionnaire as 79 other countries. Its standard measure evaluates pregnancy intention from the question: “At the time you became pregnant, did you want to become pregnant then, did you want to wait until later, or did you not want to have any (more) children at all?” [4]. The answers are categorized as “intended”, “mistimed” and “unwanted”, with “mistimed” and “unwanted” being combined to form estimates of unintended pregnancy.

In more recent years, however, it has been realized that the concept of unintended pregnancy is potentially more complex and requires more sophisticated measurement strategies [5–10]. In particular, it has been found that women’s intentions are not always clearly defined, there may be ambivalence, contradictions and doubts, and that contraceptive use and other behaviours do not always correspond to manifest pregnancy intention [11–15]. In response to the call for better measurement, two new psychometrically-valid measures of pregnancy intention were developed. The first was developed by Morin et al. [8] in Canada and the other by Barrett et al. [10] in United Kingdom, called the *London Measure of Unplanned Pregnancy* (LMUP). The Canadian measure [8], even though it presents reasonable psychometric properties and can be used to measure different grades of pregnancy planning, presents long response options which would be a barrier in a poorly educated population such as some sections of the Brazilian female population. In contrast, the LMUP is a short and self-administered measure, comprising six items to measure only one construct: pregnancy planning/intention (its original version in English is available at www.lmup.org.uk). Through the six questions (relating to contraceptive use, timing of motherhood, intention, desire for a baby, discussion with a partner, and pre-conceptual preparation), the LMUP scores pregnancy intention on a continuous scale from zero to 12 with each increase in score representing an increase in the degree of pregnancy intention. The advantages of the LMUP are that it is short, easy to complete, and can be applied to any pregnancy regardless of outcome (i.e. birth, miscarriage, abortion). Also, it makes no assumption about the nature of women’s relationships and does not assume a particular form of family building. This measure has not been validated into Portuguese language yet, although its translation and validation were successfully concluded in other countries [16–20].

As existing measures developed in specific contexts need to be evaluated before use in new ones [21] and because there are differences across populations in terms of contraceptive practice, gender relations and pregnancy expectations, our aim was to validate the LMUP for the Portuguese language, Brazilian version, and to evaluate its psychometric properties. The availability of a reliable and accurate measure of pregnancy intention in Brazil is imperative to provide relevant information on fertility related-behaviours, to understand the consequences of pregnancy intention on maternal and child health, and to determine the contexts and reasons women and couples are unable to reach their fertility goals.

### Context

In recent decades, Brazil has experienced remarkable progress in socioeconomic development, with positive consequences for the majority of its health indicators. In particular, the country has achieved improvements in its reproductive indicators, such as a high proportion of women using modern contraceptive methods, and universal access to prenatal care [22]. On the other hand, the country still needs to tackle remaining problems, like the persistent health and social inequalities, high maternal mortality rates, frequent caesarean sections, and restrictive laws around abortion [22]. In Brazil, abortion is legal only in situations of sexual violence, risk to the woman’s life, and fetal anencephaly. This means that Brazilian women who wish to terminate a pregnancy usually seek emergency care in hospitals following the use of misoprostol [23]. Women who can afford to pay for private services can access safe abortion elsewhere, though still illegal. Legal abortion is only available in a few public services around the country.

Many changes have also been observed in women’s social status in recent years, with improvements of their participation in the labor force and education, but gender equality is still a major challenge. Responsibilities for preventing pregnancy and bringing up children, for instance, are mainly considered women’s roles, especially among the lowest socioeconomic groups. This traditional social expectation of the female gender role still pushes Brazilian women towards early union and childbearing, which occur very close to each other over the ages of 20 to 24 years. For the highest socioeconomic group, however, postponement of childbearing is now a reality, as these women have high expectations around professional and educational achievements. The country experienced a rapid drop in its total fertility rate from 6.3 children per woman in 1986 to 1.9 in 2006, and there is now a strong two-child norm, with the mean ideal number of children being 2.1. The reasons for the fertility decline are numerous, but it was primarily brought about by a high contraceptive prevalence rate, with many women ending up their reproductive life with sterilization. Despite the changes in social status and the widespread use of modern contraceptive methods by the majority of Brazilian women, the proportion of pregnancies classified as unintended has not declined as expected [4, 24, 25].

## Methods

### Translation of the LMUP

For translation purposes, the English LMUP was sent to two native Brazilian Portuguese speakers (both professors at the University of Sao Paulo, with research expertise in reproductive health, and aware of the purpose and background of the LMUP) who each independently translated it into Portuguese. ALVB reviewed the translations and discussed the differences at a consensus meeting with four other health researchers – doctors and nurses (two experts in psychometrics and two in reproductive health). At the time of translation, no content of the UK version was changed. The agreed translation produced by this meeting was sent for back-translation to a native British English speaker who spoke Portuguese fluently as a second language. This person was only broadly aware of the purpose of the LMUP.

Following back-translation, we piloted the Portuguese LMUP in the form of a self-completion survey with 126 pregnant women waiting for their first prenatal care consultation in a primary health care service in a medium sized municipality in Sao Paulo state, Brazil. We asked the women, “Have you understood the questions of this questionnaire?”, with the answer options “I did not understand anything”; I understood it a bit”; “I understood almost everything, but still have some doubts”; and “I understood it perfectly well and I have no doubts”. Only one woman expressed doubts about the questions but made no suggestions for improvement. Although the majority reported they could understand it all, some women who had had low scores on items 1-5 (and therefore appeared to have an unplanned pregnancy) also reported that they had carried out a pre-pregnancy preparatory behavior (a higher score on item 6). In our initial reliability analyses we could also see that item 6 had an item-rest correlation of <0.2 and was performing differently to the other items within the scale. We were concerned that item was being miscomprehended by some women. Generally, Brazilian women seek health assistance before conception just when they notice infertility problems or when they have pre-existing diseases, such as diabetes or hypertension. For instance, pre-pregnancy folic acid use has previously been reported by only 4.3 % of Southern Brazilian women, which denotes the scarce amount of preconception care delivered in Brazil [26]. We also considered the heterogeneous schooling profile of Brazilian health system users, with many poorly educated women. Considering this context, we decided to not change the response options because we considered them clear and appropriate for Brazil. Instead, we adapted the Brazilian Portuguese LMUP for interviewer-administration, in the same way the Indian and Malawian versions did [18, 19], with changes in the wording in a way that made its administration by an interviewer possible. ALVB and FBC adjusted the phrasing and NCN and OAS checked the new interviewer-administered version, which satisfactorily addressed this issue.

### Study participants

The interviewer-administered version of the Brazilian Portuguese LMUP was field-tested at three settings in Sao Paulo City, corresponding to three different studies. The first setting (setting 1) was a public maternity hospital, where all adolescent women waiting for prenatal care (irrespective of pregnancy trimester) or hospitalized the day after giving birth in June 2012 were invited to participate in the study (*n* = 76). The purpose of this study was simply to validate the Brazilian Portuguese LMUP among adolescents.

The second setting (setting 2) comprised 12 public primary health care services. Every pregnant woman, also irrespective of pregnancy trimester, waiting for prenatal care, vaccination, or educational group meetings on specific weekdays from April to July 2013 was invited to participate in the study (*n* = 513). The purpose was to understand the reasons women with unplanned pregnancy had not used emergency contraception to prevent the pregnancy [27].

The third setting (setting 3) was another public maternity unit. Women hospitalized due to incomplete abortion – spontaneous or induced – were invited to participate in the study after emergency treatment, while waiting for hospital discharge, from May to August 2012 (*n* = 170). Having a setting with women who had an incomplete abortion provided us with two conditions essential to testing LMUP scale: first, a pregnancy outcome that is not a birth; second, a group of women who would include some with low pregnancy intention, as this group consisted of women with spontaneous and induced abortion. We did not ask women if the abortion was induced as we recognized it would be inaccurately reported due to the restrictive abortion laws and therefore underestimated. Women who responded to the LMUP at this setting were then followed-up for six months to understand their contraceptive behavior and its relation to primary health care [23].

Although we had no refusals in settings 1 and 2, we had 14 refusals in setting 3. Considering the restrictive laws on abortion in Brazil, we believe some women might have felt apprehensive taking part in this study while they were hospitalized for post-abortion care.

### Data collection

Nurses and midwives, who were graduate students, conducted all the interviews after training. Women were interviewed in quiet places in the clinics. In each setting, all pregnant/postpartum women/women who had an abortion attending the services were invited to participate in the study. In addition to the LMUP, women answered structured questions about their demographic and reproductive characteristics.

### Data analysis

We entered data twice on Epiinfo software and corrected typing mistakes. The analysis of psychometric properties were conducted with Stata 13.0 using a Classical Test Theory-based approach to facilitate comparison with the original UK study [10] and previous validations [16–20].

Rates of missing data were assessed to give an indication of items that might have a problem with acceptability or comprehension. To assess item discrimination the item-endorsement values were examined to see if any item response option had an endorsement greater than 80 %. The distribution of total scores was considered to see if the full range of scores were present and to evaluate the targeting of the scale.

To assess reliability, internal consistency was evaluated by calculating the Cronbach’s α statistic (>0.7 indicating acceptable reliability) [28] and the item-rest correlations (<0.2 indicating that the item is contributing little to the homogeneity of the scale) [21]. We also examined the inter-item correlations, checking that all correlations were positive.

Consistent with the UK LMUP development and evaluation study [10], construct validity was examined using two methods: principal component analysis and hypothesis testing. We used principal component analysis (without rotation, requesting as many components as there were Eigenvalues >1) to test the hypothesis that all items would load onto one component (i.e. measure the same construct). Hypotheses related to pregnancy intention were generated from information provided by the Brazil DHS 2006. We tested four hypotheses: 1) women in the extreme age groups (younger and older) would have lower levels of pregnancy intention/lower LMUP scores; 2) women from lower educational backgrounds would have lower pregnancy intention/lower LMUP scores; 3) those who are not married would have lower intention/lower LMUP scores; and 4) women from the third setting (i.e. those who were hospitalized for incomplete abortion and therefore likely to include some with induced abortions) would have lower intention/lower LMUP scores compared with the other two centres. We used the Wilcoxon Rank-Sum test and the Mann Whitney *U* test to assess significance.

Finally, in keeping with the USA evaluation of the LMUP [17], we carried out an exploratory analysis based on the principles of modern test theory: a Mokken scaling procedure (monotone homogeneity assumption). Mokken models are non-parametric models within the family of Item Response Theory models. The results of the Mokken analysis allowed us to see whether the items conformed to a probalistic Guttman structure, i.e. that items vary in ‘difficulty’, some being easy to endorse, some being more difficult to endorse, and that respondents who have a particular level of the construct (in this case pregnancy planning/intention) should broadly endorse items up to the level of their construct and then not endorse items beyond that. The Loevinger H coefficient produced by the Mokken analysis relates to Guttman errors, with a lower H value indicating more observed Guttman errors. Items with a Loevinger H coefficient >0.3 were eligible for scaling [29, 30]. The scale as a whole was also assessed by a Loevinger H coefficient, with <0.4 meaning the scale is “weak”, 0.4 to 0.49 meaning the scale is “medium”, and >0.5 meaning the scale is “strong” [29]. The construction of an adequate Mokken scale confirms that the raw score can be used to order respondents on the construct being measured [30].

## Results

### Samples

The field test sample comprised 759 women aged 15 to 44. Their socio-demographic and reproductive characteristics are shown in Table 1. The majority of women were married and were, on average, five years younger than their partners.

**Table 1:** Socio-demographic and reproductive characteristics of women in the field test

Characteristics	Mean	Sd
Age (years)	25.9	7.1
Partner age (years)	30.1	8.0
Menarche (years)	12.7	1.6
Age at first intercourse (years)	16.5	2.6
Age at first pregnancy (years)	20.6	4.9
Number of children	1.0	1.2
Education (years)	9.4	2.5
	N	%
Married	600	79.1
Work paid job	378	49.8
First pregnancy	306	40.3
Age group		
15–19	188	24.8
20–24	174	22.9
25–29	163	21.5
30–34	122	16.1
35–39	85	11.2
40+	27	3.6
Education		
Low (0 to 9 years of schooling)	259	34.1
Mid (10 to 12 years of schooling)	450	59.3
High (13 or more years of schooling)	50	6.6
Status at interview		
Pregnant	524	69.0
Post-abortion	170	22.4
Postpartum	65	8.6

### Acceptability and targeting

**Fig. 1:**
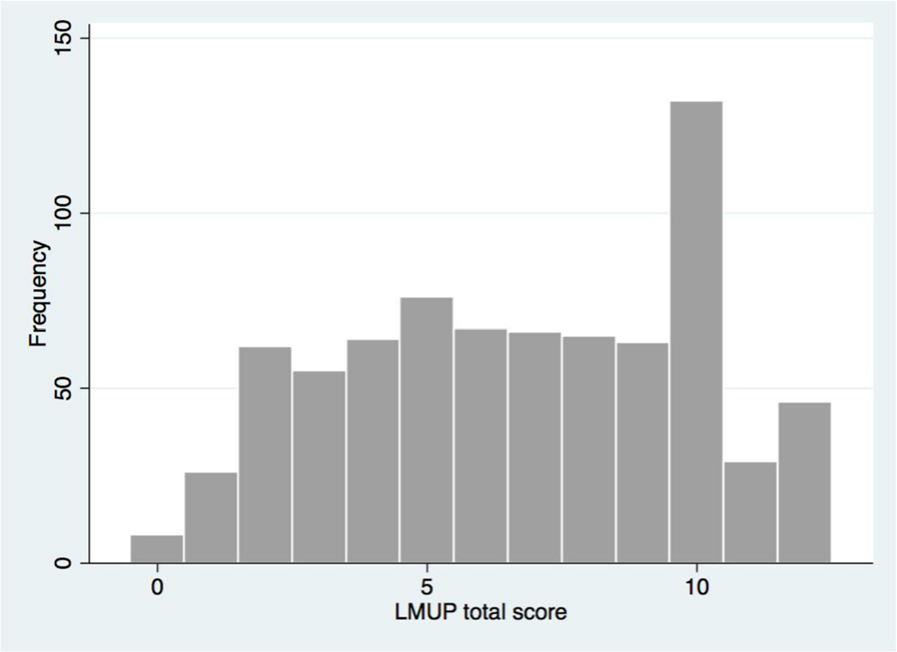
Distribution of LMUP total scores

We did not observe any missing data. One question had a response option with more than 80 % endorsement, which was the item concerning preparation (item 6, category 0: did no preparatory behaviors) (Table 2). We also found the full range of LMUP scores (Fig. 1). The LMUP score distribution was non-Normal. The median score was 7 (inter-quartile range 4–10), with 19.9 % of women scoring 0–3 (unplanned); 52.3 % scoring 4–9 (ambivalent); and 27.8 % scoring 10–12 (planned).

**Table 2:** Endorsement of the LMUP items and response options

Item	Category	N	%
1. Contraception	0. always using contraception	79	10.4
	1. using sometimes or failed at least once	192	25.3
	2. not using contraception	488	64.3
2. Timing	0. did not want pregnancy at all	156	20.6
	1. wanted pregnancy later	272	35.8
	2. wanted pregnancy then or sooner	331	43.6
3. Intention	0. did not intend pregnancy	291	38.3
	1. intentions kept changing	147	19.4
	2. intended pregnancy	321	42.3
4. Desire	0. did not want baby	172	22.6
	1. mixed feelings about having baby	125	16.5
	2. wanted baby	462	60.9
5. Partner	0. never discussed getting pregnant	104	13.7
	1. discussed but not agreed to get pregnant	303	39.9
	2. agreed to get pregnant	352	46.4
6. Preparation	0. did no preparatory behaviors	640	84.3
	1. did 1 preparatory behavior	55	7.3
	2. did 2 or more preparatory behaviors	64	8.4
Total		759	100,0

### Reliability

The Cronbach’s alpha was 0.813 and all the item-rest correlations were above 0.20 (item 1: 0.413; item 2: 0.662; item 3: 0.724; item 4: 0.645; item 5: 0.614; item 6: 0.394). All the inter-item correlations were positive, and showed moderate to strong correlations between items (they ranged from 0.238 to 0.620). Principal component analysis confirmed that all six items loaded onto one component (Eigenvalue = 3.12), with all component loadings greater than 0.5 (item 1: 0.555, item 2: 0.795, item 3: 0.842, item 4: 0.783, item 5: 0.754, item 6: 0.538).

### Validity

The results of hypothesis testing showed that all construct validity hypotheses were confirmed. Lower LMUP median scores were observed in the extreme age groups, such as 15-19 (median = 6) and 40 and more (median = 5), compared to the other groups, whose medians ranged from 7 to 8 (*p* <0.001). Non-married women (*p* = <0.001) and women with lower educational backgrounds also had the lowest median LMUP scores (5 and 6, respectively) compared to their married (median = 8) and middle and high educational background counterparts (median = 7 and 8, respectively) (*p* <0.001). Also, analysis of LMUP score by setting showed that setting 3 had a significantly lower LMUP score (median = 6) compared to the other centres (median = 7) (*p* = 0.020).

### Scaling

The Mokken analysis showed that items varied in their ‘difficulty’, with item 1 being easiest to endorse, followed by items 5, 2, 4, and 3, and item 6 as hardest to endorse. The items conformed to a basic Guttman structure (Loevinger H values: item 1, 0.42; item 2, 0.60; item 3, 0.65; item 4, 0.61; item 5, 0.57; item 6, 0.85). The Mokken scaling procedure selected all items for the scale, with an overall Loevinger H coefficient of 0.60.

## Discussion

We validated the LMUP for the Portuguese language, Brazilian version. Our results indicate that the Brazilian Portuguese LMUP performed extremely well, with demonstrated reliability and validity in terms of acceptability, endorsement, targeting, internal consistency, construct validity, and scaling.

There was only one psychometric criterion on which the Brazilian Portuguese LMUP did not perform quite so well: endorsement. Endorsement showed that a very high proportion (84 %) of women scored 0 on item 6, i.e. they reported that they did not carry out a pre-pregnancy preparatory activity. This is a minor issue as the item did discriminate between women and there were no other issues of targeting with the scale. The endorsement pattern of item 6 is likely to be an accurate reflection of the low level of pre-conceptual preparatory activity in Brazil, similar to that described in Iran [20]. Preconception health is not part of routine care in the country’s national health system yet, so Brazilian women are not familiar with taking preparatory behaviours in order to better prepare for pregnancy. Pre-conception care may become a priority in Brazil primary health care policies and programs in future, as its implementation has positive impacts on maternal and newborn health [31]. It is likely that as pre-conceptual care programs are implemented, the endorsement pattern of item 6 will change in response.

Compared to the original UK LMUP [10] and the versions in USA [17], India [18], Malawi [19], and Iran [20] our Brazilian Portuguese version of the LMUP performed comparably or better (Table 3). Like the original UK measure and other translated versions, the internal consistency was excellent. In terms of construct validity, the Brazilian Portuguese version of the LMUP clearly measured one construct and, unlike the Indian and Malawi versions, experienced no issues with item 1 (contraception) potentially measuring another construct.

**Table 3:** Comparison of the validation results of the original LMUP and its translations

	Internal consistency Cronbach’s alpha	Test-restest Weighted Kappa	Construct validity Principal Component Analysis (Eigenvalues)	Scaling Loevinger H
UK – English	0.92	0.97	4.33	-
USA – English	0.78	0.72	2.9	0.53
USA – Spanish	0.84	0.77	3.4	
India – Kannada	0.76	0.43	2.66 and 1.05	-
India – Tamil	0.71			-
Malawi – Chichewa	0.78	0.80	3.1 and 1.00	-
Iran – Persian	0.87	-	-	-
Brazil – Portuguese	0.81	-	3.12	0.60

With scaling, all items were selected into the scale, with a Loevinger H value indicating the scale was strong, confirming that the LMUP score can be used to order women along the continuum of the pregnancy planning/intention. In comparison, scaling of the USA version of the LMUP did not initially include item 1 (contraception) as it fell short of the threshold for inclusion, although the USA LMUP was still a strong scale with the inclusion of item 1.

In other contexts, it has been suggested that item 1 might have been understood as the use of modern contraceptive methods [17, 18]. Since the use of contraception in Brazil is high and concentrated in modern methods, like the pill (29 %) and sterilization (29 %), with a recent increase in the use of injectables [4], we may consider that women in this study may not have found difficulties when answering this item. In that way, although Brazil is not a high-income country and could be comparable to India in terms of social inequalities and educational background, Brazilian women seem to have contraceptive practices closer to some Western countries, such as the USA and UK, than other low and mid-income countries, especially from the sub-Saharan Africa and Asia.

There were some limitations of this study. The first limitation resulted from the legal restrictions on abortion in the country, which precluded us distinguishing the effects of an induced or spontaneous abortion on the scale reliability. However, our analysis of LMUP scores by setting confirmed that the setting with women who had an abortion had significantly lower median. The second limitation was that we interviewed only Brazilian public health system users. As this is a universal health system, the entire population can be considered a system user, but exclusive users tend to report lower incomes and have a lower educational level compared to women attending private clinics. Nevertheless, our sample was heterogeneous in terms of women’s educational background, considering women with no schooling at all up to women with tertiary education. The third limitation was in relation to the psychometric analyses that we carried out: we included no test-retest (which looks at the reliability of the scores in terms of their stability, normally over a short term period such as two weeks). Also, we were unable to carry out a longer term test-retest, comparing the LMUP scores reported by women when they were pregnant with after birth, a test which has been carried out in other evaluations of the LMUP. These aspects of reliability of the Brazilian Portuguese LMUP still require investigation.

The strengths of our study included our large sample size and the fact that we included pregnant women of all trimesters, postpartum women and women who had an abortion (spontaneous or induced), in other words, all possibilities of pregnancy outcomes.

## Conclusion

The Brazilian Portuguese LMUP is a valid and reliable measure of pregnancy planning/intention that is now available for use in Brazil. As a psychometrically-validated outcome measure of pregnancy intention/planning, it represents a useful addition to the public health research and surveillance toolkit in Brazil, and a methodological advance on the DHS questions relating to pregnancy intention. Further research, however, is recommended on the stability of reporting of pregnancy intention in Brazil.

## Abbreviations

DHS: Demographic and Health Survey
LMUP: London Measure of Unplanned Pregnancy
SD: standard deviation
UK: United Kingdom
USA: United States of America
